# BtuB-Dependent Infection of the T5-like *Yersinia* Phage ϕR2-01

**DOI:** 10.3390/v13112171

**Published:** 2021-10-28

**Authors:** Lotta J. Happonen, Maria I. Pajunen, Jin Woo Jun, Mikael Skurnik

**Affiliations:** 1 Division of Infection Medicine, Department of Clinical Sciences Lund, Lund University, 22184 Lund, Sweden; lotta.happonen@med.lu.se; 2 Department of Bacteriology and Immunology, Medicum, Human Microbiome Research Program, Faculty of Medicine, University of Helsinki, 00290 Helsinki, Finland; maria.pajunen@helsinki.fi; 3 Department of Aquaculture, Korea National College of Agriculture and Fisheries, Jeonju 54874, Korea; advancewoo@snu.ac.kr; 4 Division of Clinical Microbiology, HUSLAB, University of Helsinki and Helsinki University Hospital, 00290 Helsinki, Finland

**Keywords:** bacteriophage, phage ϕR2-01, *Yersinia enterocolitica*, *Escherichia* phage T5, BtuB, genome, proteome

## Abstract

*Yersinia enterocolitica* is a food-borne Gram-negative pathogen responsible for several gastrointestinal disorders. Host-specific lytic bacteriophages have been increasingly used recently as an alternative or complementary treatment to combat bacterial infections, especially when antibiotics fail. Here, we describe the proteogenomic characterization and host receptor identification of the siphovirus vB_YenS_ϕR2-01 (in short, ϕR2-01) that infects strains of several *Yersinia enterocolitica* serotypes. The ϕR2-01 genome contains 154 predicted genes, 117 of which encode products that are homologous to those of *Escherichia* bacteriophage T5. The ϕR2-01 and T5 genomes are largely syntenic, with the major differences residing in areas encoding hypothetical ϕR2-01 proteins. Label-free mass-spectrometry-based proteomics confirmed the expression of 90 of the ϕR2-01 genes, with 88 of these being either phage particle structural or phage-particle-associated proteins. In vitro transposon-based host mutagenesis and ϕR2-01 adsorption experiments identified the outer membrane vitamin B12 receptor BtuB as the host receptor. This study provides a proteogenomic characterization of a T5-type bacteriophage and identifies specific *Y. enterocolitica* strains sensitive to infection with possible future applications of ϕR2-01 as a food biocontrol or phage therapy agent.

## 1. Introduction

*Yersinia enterocolitica* is a zoonotic, food-borne Gram-negative bacterium of the family *Enterobacteriaceae* that can cause yersiniosis in humans and animals [1]. The predominant symptom in humans is gastroenteritis [2]. The main animal reservoir for *Y. enterocolitica* is pigs, and pork-derived products are thought to be the main source of human infections, in addition to blood transfusions and intake of contaminated drinking water [1,2]. In addition to pigs, *Y. enterocolitica* can be found in sewage water [3,4]. Several bacteriophages that infect *Y. enterocolitica* have previously been isolated from the raw incoming sewage of city sewage treatment plants in Finland and from pig stool samples [5,6,7]. These phages were isolated using different host strains for enrichment. Many of these bacteriophages use different parts of the *Y. enterocolitica* lipopolysaccharide (LPS) as the receptor [5,7]. Detailed characterization of several of these bacteriophages has been reported earlier, for example, the T3-related ϕYeO3-12 [8,9,10], the jumbo phage ϕR1-37 [11,12,13], the T4-like phage ϕR1-RT [14], and the podovirus ϕ80-18 [15].

Bacteriophage T5 is a lytic phage that infects *Escherichia coli*, and a type member for the T5-like *Siphoviridae* family that infects Gram-negative bacteria [16,17]. The T5 genome is 121,752 bp in size, including 10,139 bp identical terminal repeats (TRs), and it contains a total of 168 predicted proteins and 24 tRNA coding genes [16]. Mass spectrometric methods have identified 16 phage-particle-associated proteins (PPAPs) ([17] and references therein). Functions have been associated with 61 (36.3%) of the predicted T5 gene products based on their similarity to other gene products and they are mainly involved in phage DNA replication and repair, nucleotide metabolism, and host cell lysis, as well as being structural phage proteins [16]. The T5 genes are arranged in three clusters. The first cluster is composed of 17 pre-early genes encoding proteins with presumed functions in the inhibition of inactivating host functions [16,18]. The second cluster is formed by 111 early genes encoding proteins with functions in DNA replication, recombination, repair, and transcription, proteins involved in phage lytic processes, and 24 tRNA encoding genes [16]. Lastly, the third cluster contains 23 late genes, encoding mainly for phage structural proteins and proteins involved in phage morphogenesis [16,17]. During the infection, T5 attaches to *E. coli* in a two-step manner: first, by reversible binding to the lipopolysaccharide (LPS) O-antigen mediated by the L-shaped tail fibers, followed by a second, irreversible step, in which the T5-receptor-binding protein (Gp5) binds to the host’s outer membrane iron-ferrichrome transporter FhuA [19,20], leading to a lytic infection cycle.

Lytic phages are powerful tools as phage therapy and biocontrol agents [21,22]. In response to the increasing global anti-microbial resistance (AMR), phage therapy—the use of lytic bacteriophages to cure patients with bacterial infections—is gaining a renewed interest in the Western world after being largely abandoned in the 1940s [23]. Phage therapy offers an alternative to antibiotics as phages infect and also kill AMR bacteria. Phages very often possess narrow host specificity, each phage infecting only a few bacterial species or strains, leaving the normal microbiota unharmed. The study of bacteriophages provides insight into phage genome evolution, protein expression, and bacterial adaptation to phage infections, and could promote the development of novel phage-based biotechnological products [22]. For *Y. enterocolitica*, some bacteriophages have already been described with potential as biocontrol agents to reduce the number of colonies in meat [24], food and kitchenware [6], and poultry [25]. This is especially essential for the psychrophilic *Y. enterocolitica,* which is able to proliferate at 4 °C, making it dangerous even when contaminated food products are stored under refrigeration. Even though the regulatory issues regarding the use of phages as biocontrol agents in the food industry is a major obstacle, they have been used for this purpose since 2006 [26,27].

Here, we describe the proteogenomic and morphological characterization of phage ϕR2-01 isolated from the incoming sewage of the sewage treatment plant of Turku, Finland, based on its ability to infect the rough *Y. enterocolitica* serotype O:8 strain 8081-c-R2 [5]. We present evidence that ϕR2-01 uses the host outer membrane protein BtuB as a receptor, while calcium and the host LPS have little or no effect on the phage infectivity. This adds phage ϕR2-01 to the group of phages targeting BtuB for host infection.

## 2. Materials and Methods

### 2.1. Bacterial Strains, Phage Isolation, and Growth Conditions

Bacteriophage ϕR2-01 was isolated in 1998 from the incoming sewage water of the City of Turku, Finland, as described for other *Yersinia* phages [5], using as a host the rough *Y. enterocolitica* serotype O:8 strain 8081-c-R2 [28] (Table 1). The isolation protocol of phage particles for DNA extraction and genome sequencing was carried out using standard laboratory protocols [29]. To prepare phage particles for electron microscopy, the ϕR2-01 lysate was centrifuged to remove bacterial debris, and the supernatant was treated with chloroform and concentrated by ultrafiltration with Amicon Ultra-4 (30 kDa) centrifugal filter units (Merck KGaA, Darmstadt, Germany). The concentrated phage suspension was further purified by rate-zonal centrifugation on a linear 15–35% glycerol gradient, as described in [11].

### 2.2. Host Range Determination

Host range determination of the phage was carried out using the soft agar overlay method. The bacteria were grown to an OD_600_ of ~1, and 100 µL of the suspension was mixed with 3 mL of melted 0.4% agar (adjusted to 50 °C) and immediately poured on top of a Luria agar plate. After solidification of the soft agar, 10 µL drops of 10-fold serial dilutions of the phage stock were applied on top of the soft agar and the plates were incubated at 22 °C. The lysis zones were evaluated the following day. On selected strains, the double-agar overlay titration method was used to calculate more accurately the efficiencies of plating (EOP).

### 2.3. Genome Sequencing, Assembly, and Annotation

The ϕR2-01 genome was sequenced using Illumina GAIIx (Genome Analyzer) technology at the FIMM Sequencing unit (Helsinki, Finland). The sequence data yielded 7 contigs, of which one was >104 kpb, and the others between 0.2 and 5.5 kpb. Most of these contigs showed high similarity to bacteriophage T5 and could thus be aligned with the T5 genome (GenBank no. NC_005859) [16]. This allowed us to design PCR primers (ϕR2-01-R1, ϕR2-01-R4, ϕR2-01-R5, and ϕR2-01-F5, Table S1) to amplify the gap sequences between the contigs and subsequently sequence the PCR products. Sequence assembly and analysis were done with the Staden software package [39]. The total length of the joined contigs was 112,795 bp. The T5-type terminal repeats were identified by realigning the sequence reads against the 112,795 bp genome using the TopHat read aligner [40]. That allowed the identification of the terminal repeats based on the region with duplicated read coverage of the genome. Average read coverage over the whole genome was ~1000 and ~2000 over the terminal repeat. The identification and prediction of genes was done using the Artemis tool [41] and the RAST service [42]. The PSI-BLAST [43] program (https://blast.ncbi.nlm.nih.gov/Blast.cgi (accessed on 12 June 2021)) was used to identify homologous proteins. Genome identity analysis between different viruses was carried out using EMBOSS StretcherN at EBI [44].

### 2.4. Electron Microscopy

For electron microscopy, ϕR2-01 was propagated on 8081-c-R2 (Table 1). The ϕR2-01 lysate was centrifuged to remove bacterial debris, and the supernatant was treated with chloroform and concentrated by ultrafiltration with Amicon Ultra-4 (30 kDa) centrifugal filter units (Merck KGaA, Darmstadt, Germany). The concentrated phage suspension was further purified by rate-zonal centrifugation on a linear 15–35% glycerol gradient, as described in [11]. We obtained two light-scattering bands in the glycerol gradient, of which the lower band was used for microscopy based on purity estimation by SDS-PAGE (not shown). The lower phage-containing band was further concentrated and buffer-exchanged into a TM buffer (50 mM of Tris-HCl with a pH of 7.8 and 10 mM of Mg_2_SO_4_) using Amicon Ultra-4 (30 kDa) centrifugal filter units prior to negative staining electron microscopy. The samples for negative staining electron microscopy were prepared essentially as described in [45]. Briefly, 5 μL aliquots of phage ϕR2-01 were adsorbed on holey-carbon film-coated grids (Quantifoil R 2/2) for 1 min prior to negative staining with 2% (wt/vol) uranyl acetate (pH 4.5). The phages were imaged in a FEI F20 field emission gun transmission electron microscope (FEI, Eindhoven, the Netherlands) operating at 200 kV. Phage-particle-containing micrographs were recorded on a Gatan UltraScan 4000 charge-coupled device (CCD) camera (Gatan, Inc., Pleasanton, CA, USA) at nominal magnifications of 39,440× and 68,000×. The data were collected in the Biocenter Finland National Cryo-EM unit, Institute of Biotechnology, University of Helsinki (Helsinki, Finland).

### 2.5. Isolation of Phage-Resistant Mutants

A previously generated in vitro transposon insertion mutant library of YeO3-R1 [46] was used to isolate phage-resistant mutants. A fresh aliquot of the YeO3-R1::Cat-Mu library was grown in LB with chloramphenicol (20 μg/mL) at RT to a mid-logarithmic phase. To a 1 mL aliquot of bacteria, ϕR2-01 phage was added at MOI > 1 to allow infection at high multiplicity. After 3 min, 9 mL of media was added and the bacteria were grown for 4 h. Subsequently the bacteria were washed two times with LB, diluted, and plated on Yersinia-selective agar (CIN agar, cefsulodin–irgasan–novobiocin agar) with chloramphenicol. Twenty colonies were streaked and retested for phage resistance: 14 colonies were resistant, 4 colonies were uncertain due to poor growth, and 2 were sensitive.

### 2.6. Identification of Transposon Insertion Sites

The Cat-Mu transposon insertion sites were identified by sequencing a PCR fragment produced by arbitrary PCR. First, bacterial genomic DNA from 10 resistant clones was isolated from an overnight culture using a JetFlex Genomic DNA Purification Kit (Thermo Fisher Scientific, Waltham, MA, USA). The arbitrary PCR was performed in two steps as follows. In the first step, 250 ng of gDNA was used as a template in PCR reactions with primers Muc2 and Arb1, 0.5 µM each (Table S1), 0.4 U DyNAzyme II DNA polymerase, 1× Optimized DyNAzyme II Buffer (with 1.5 mM of MgCl_2_), and dNTPs (0.2 mM each). The following conditions were used for the first PCR: initial denaturation of 5 min at 95 °C, 6 cycles (30 s at 95 °C, 30 s at 30 °C, and 1.5 min at 72 °C), followed by 30 cycles (30 s at 95 °C, 30 s at 45 °C, and 2 min at 72 °C), and a final extension of 5 min at 72 °C. The second round of PCR with primers Arb2 and MucInt (Table S1) was carried out using the PCR product obtained from the first PCR as a template (1 µL); other reagents were the same as above. The following conditions were used for the second nested PCR: initial denaturation of 5 min at 95 °C and 30 cycles (30 s at 95 °C, 30 s at 45 °C, and 2 min at 72 °C). As a negative control, wt YeO3-R1 gDNA was used. The PCR products were analyzed with 1.5% agarose gel electrophoresis, and the most prominent bands from 4 resistant clones (10R, 11R, 15R, and 20R) (Table 1) were selected and purified from preparative agarose gels and then sequenced using MucInt as a sequencing primer.

### 2.7. Complementation of the Y. enterocolitica O:3 BtuB Mutant

The full open reading frame (ORF) of the *btuB* gene plus the upstream promoter region of *Y. enterocolitica* serotype O:3 strain YeO3-c was cloned as a 2.34 kb PCR fragment. The fragment, amplified with Phusion DNA Polymerase using the primer pair BtuB-F1 and BtuB-R1 (Table S1), was cloned into the suicide vector pSW25T to obtain plasmids of pSW25T_BtuB (Figure S1) to be used in single-copy in trans complementation. In addition, the *btuB* gene was cloned into plasmid pTM100 to be used for overexpression complementation. Briefly, the 2.34 kb PCR fragment was digested with *Mfe*I and ligated with *Eco*RI-digested, CIP-treated pTM100 or pSW25T. The constructed plasmids were mobilized from the *E. coli* host to the *btuB* mutants YeO3-R1-11R and YeO3-R1-15R by diparental conjugation, as described earlier [34].

### 2.8. Sequencing of the Yersinia BtuB

The *btuB* ORF of naturally phage-resistant *Y. enterocolitica* serotypes and serotypes that had both sensitive and resistant strains (Table 2 and Table S2) were PCR amplified with Phusion DNA Polymerase using the primer pair BtuB-F1 and BtuB-R1 (Table S1). In addition, *Y. kristensenii* serotype O:3 and control strains were included in the analysis. The PCR fragments were Sanger-sequenced at the FIMM Sequencing unit (Helsinki, Finland) with the PCR primers and four additional internal sequencing oligonucleotides (Table S1). The sequences of the *btuB* genes of *Yersinia* strains sequenced in this work have been deposited to GenBank under the accession numbers OK169486-OK169508.

### 2.9. Phage Adsorption Assay

Approximately 5 × 10^3^ PFU of ϕR2-01 in 100 µL was mixed with a 400 µL sample of bacteria (*A*_600_ 1.2) in multiple replicates, and the suspensions were incubated at RT for 12 to 87 min. At various time points, replicate tubes were withdrawn and centrifuged at 16,000× *g* for 3 min. The total adsorption time is achieved by adding the centrifugation time to the time of sampling. The phage titer remaining in the supernatant, i.e., the residual PFU percentage, was determined by plating 100 µL aliquots. LB was used as a non-adsorbing control in each assay, and the phage titer in the control supernatant was set to 100%. To study the effect of calcium in adsorption, LB was supplemented with 1 mM CaCl_2_. Each assay time point was performed in duplicate and repeated at least three times.

### 2.10. Sample Preparation for Mass Spectrometry

For mass spectrometry, 5 μL of concentrated phage lysate (6 × 10^10^ PFU/mL; protein concentration determined as 2.5 mg/mL using a Pierce BCA kit) and purified phage particles (2 × 10^12^ PFU/mL; protein concentration of 2.8 mg/mL) were used for trypsin digestion. The samples were prepared for mass spectrometry essentially as described in [45]. Phage samples were mixed with 8 M urea–100 mM ammonium bicarbonate to a final volume of 50 μL, and the cysteine bonds were reduced with 5 mM tris(2-carboxyethyl)phosphine (TCEP) (37 °C for 60 min) with subsequent alkylation using 10 mM iodoacetamide (22 °C for 30 min). Ammonium bicarbonate, at 100 mM, was used to dilute the urea concentration of the samples to 1.5 M. Proteins were digested for 18 h at 37 °C with sequencing grade trypsin (Promega). Formic acid (10%) was used to lower the pH of the samples to 3.0, and the peptides were subsequently purified with C18 reverse-phase spin columns according to the manufacturer’s instructions (Microspin Columns, Harvard Apparatus). The dried peptides were reconstituted in 2% acetonitrile and 0.2% formic acid prior to mass spectrometric analyses.

### 2.11. Liquid Chromatography Mass Spectrometry and Protein Identification

Liquid chromatography mass spectrometry was essentially performed as described earlier [45]. A Q Exactive Plus mass spectrometer (Thermo Scientific) connected to an EASY-nLC 1000 ultra-high-performance liquid chromatography system (Thermo Scientific) was used to analyze the peptides. An EASY-Spray column (Thermo Scientific; ID 75 μm × 25 cm, column temperature of 45 °C) was used for peptide separation. The column was equilibrated and the samples were loaded using a constant pressure of 600 bars. A linear gradient of 5 to 35% acetonitrile in aqueous 0.1% formic acid for 90 min at a flow rate of 300 nL min^−1^ was used to separate the peptides. One full MS scan (resolution of 70,000 at 200 *m*/*z*; mass range of 400–1600 *m*/*z*) was followed by MS/MS scans (resolution of 17,500 at 200 *m*/*z*) of the 15 most abundant ion signals. An isolation width of 2 *m*/*z* was used to isolate the precursor ions that were then fragmented using high-energy collision-induced dissociation at a normalized collision energy of 30. Charge-state screening was enabled, and precursors with an unknown charge state and singly charged ions were rejected. The automatic gain control was set to 1 × 10^6^ for both MS and MS/MS with ion accumulation times of 100 ms and 60 ms, respectively. The intensity threshold for precursor ion selection was set to 1.7 × 10^4^.

### 2.12. Mass Spectrometry Data Analysis

The mass spectrometric data were analyzed essentially as described in [45]. MS raw data were converted to gzipped and Numpressed mzML [47] using the tool msconvert from the ProteoWizard v3.0.5930 suite [48]. The search engine X! Tandem [49] (2013.06.15.1-LabKey, Insilicos, ISB) was used to analyze the acquired spectra against an in-house compiled dataset containing the *Yersinia enterocolitica* serotype O:8/biotype 1B (strain NCTC 13174/8081) and Yersinia phage ϕR2-01 reference proteomes (UniProt proteome IDs UP000000642 and UP000002908, respectively) (both accessed on 8 June 2021), yielding a total of 4178 protein entries and an equal amount of reverse decoy sequences. We also performed an additional analysis in order to identify any expressed open reading frames missed in the genome annotation. For this, the genome of ϕR2-01 (accession number: HE956708.2) was analyzed for open reading frames via the NCBI ORF finder tool (https://www.ncbi.nlm.nih.gov/orffinder/ (accessed on 12 June 2021)) using 75 nt as the minimal ORF length, standard genetic code as code, and ATG as well as alternative initiation codons as the ORF start codon. This approach generated 542 translated ORFs, which were used together with an equal amount of reverse decoy sequences as an alternative reference proteome. Fully tryptic digestion was used allowing 1 missed cleavage. Carbamidomethylation (C) was set to static and oxidation (M) to variable modifications, respectively. Mass tolerance for precursor ions was set to 20 ppm, and for fragment ions to 50 ppm. Identified peptides were processed and analyzed through the Trans-Proteomic Pipeline (TPP v4.7 POLAR VORTEX rev 0, Build 201403121010) using PeptideProphet [50] and ProteinProphet [51] scoring. The protein false discovery rate (FDR) was set to 1% in ProteinProphet. Fraggle [52] (version 2.10.3) was used for label-free spectral counting. A protein was considered identified if it was detected in all three replicates, and the average spectral count was 2 or above.

## 3. Results and Discussion

### 3.1. ϕR2-01 Is a T5-like Siphovirus

**Figure 1:**
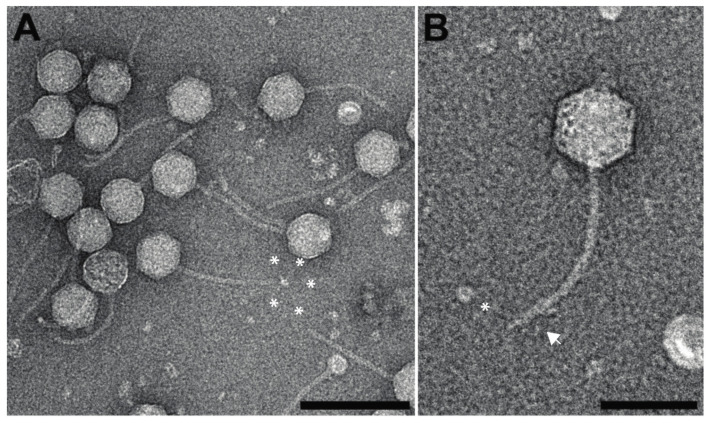
Negative staining electron micrograph of ϕR2-01. (**A**) ϕR2-01 viruses imaged at 39,440× magnification, scale bar of 200 nm. (**B**) ϕR2-01 viruses imaged at 68,000× magnification, scale bar of 100 nm. White asterisks in (**A**,**B**) point to ends of apparent tail fibers, whereas a white arrowhead in B points to a tail fiber curled up against the tail.

Based on negative staining electron microscopy, *Yersinia* phage ϕR2-01 has a siphovirus morphology with a long, non-contractile tail and an isometric capsid. The average diameter of the capsid is 82 nm edge-to-edge and 88 nm vertex-to-vertex (Figure 1), which is slightly smaller than the reported capsid size of bacteriophage T5 (94 nm vertex-to-vertex) [53]. The ϕR2-01 capsid is attached to a 185-nanometer-long tail, which is again slightly shorter than the reported 250-nanometer-long tail of T5 [53]. Based on the phage orphology and the genome similarity to bacteriophage T5 (see below), ϕR2-01 was classified as a T5-like siphovirus. The observed differences in size between ϕR2-01 and T5 might in part be explained by the fact that ϕR2-01 was imaged using negative staining microscopy, whereas the measure for T5 comes from a three-dimensional model calculated from electron cryo-micrographs [53].

### 3.2. General Genomic Features of ϕR2-01

**Figure 2:**
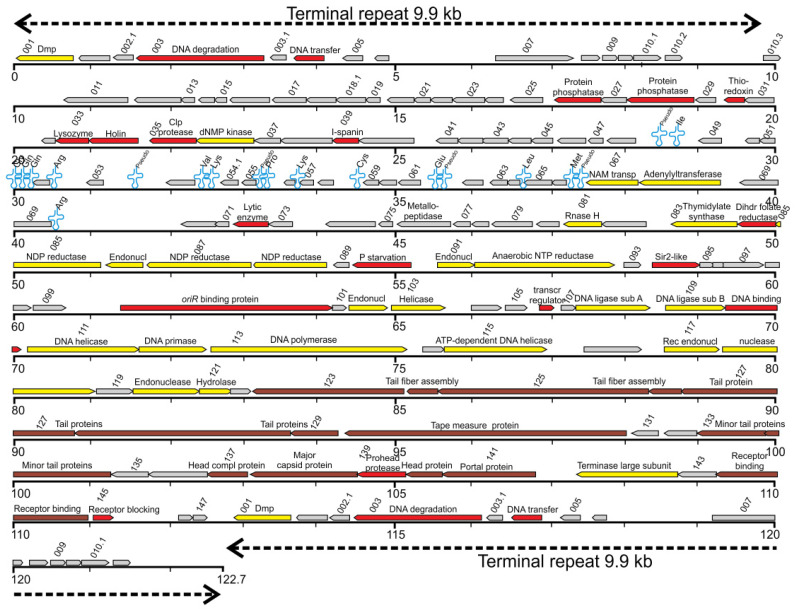
Map of the phage ϕR2-01 linear genome based on the nucleotide sequence (GenBank accession no. HE956708). The genes are shown in different colored arrows, starting with g001 at the upper left. Every second gene is indicated above the arrow, and the direction of the arrow indicates the coding direction of a given gene. Genes suggested to encode structural proteins based on homology are shown in brown, genes encoding proteins involved in nucleotide metabolism and genome replication in yellow, and genes encoding other proteins in red. The predicted functions of the gene products are indicated above the arrows. The locations of tRNA-encoding genes are shown as blue cloverleaf outlines. The terminal repeats at the genome ends are indicated with dotted lines. The figure was drawn in Artemis and modified in CorelDraw.

The linear genome of ϕR2-01 is a 122,696 bp in length double-stranded DNA genome, including 9,901 bp terminal repeats, with 154 predicted genes and 19 tRNA molecules (GenBank no. HE956708.2) (Figure 2, Table S3). The GC content of the ϕR2-01 genome is 40.2%, close to that of T5 (39.3%) [16], but notably lower than that of its host *Y. enterocolitica* 8081 (47%) (GenBank no. AM286415) [54]. The overall genome sequence identity between ϕR2-01 and bacteriophage T5 [16] is 66.4%, as determined by EMBOSS StretcherN at EBI [44]. The ϕR2-01 genome is syntenic with the T5 genome (the arrangement to pre-early, early, and late genes), with most of the differences between the phages occurring at uncharacterized ϕR2-01 genes or at ϕR2-01 genes encoding hypothetical proteins of unknown function (HPUF) (Table S3). The predicted 154 ϕR2-01 genes are encoded in six different blocks on both genome strands: three blocks (*g001-006*, *g011-g090*, and *g123-144*) on the reverse strand, and three blocks (*g007-g010.3*, *g091-122*, and *g145-147*) on the forward strand.

In total, 117 of the 154 predicted ϕR2-01 genes have unique counterparts in T5 (ten genes in ϕR2-01 are pairwise similar to five genes in T5, excluding the ones repeated in the terminal repeat region) and, in contrast, 32 of the ϕR2-01 genes are missing in T5 (Table S3). Importantly, all the morphological T5 genes seem to be conserved in ϕR2-01 (Table S3) [17,55]. In total, 105 ϕR2-01 genes—some of which are shared with T5—encode for HPUFs as determined by PSI-BLAST (Table S3). Furthermore, of the HPUF-encoding genes, four (*g010.3*, *g032*, *g056,* and *g101*) seem to be orphan genes unique to ϕR2-01 (Table S3). Notably, no known genes encoding integrases, lysogeny- or virulence-associated proteins, or acquired antimicrobial resistance genes were identified, and therefore this bacteriophage can be considered potentially safe for phage therapy.

### 3.3. Comparison of ϕR2-01 to Other Closely Related Phages

**Figure 3:**
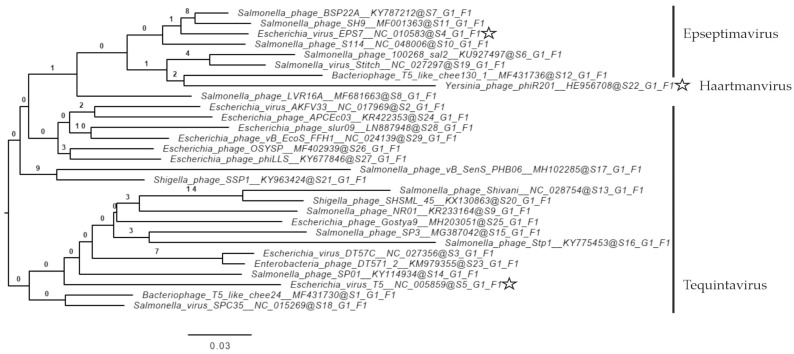
Phylogeny tree created with Victor (DSMZ) [66] (accessed on 11 October 2020). The phylogenetic GBDP tree of phage ϕR2-01, the sole member of the genus *Haartmanvirus* present in the ICTV virus taxonomy (release 2020). Other genera of the subfamily *Markadamsvirinae* are *Epseptimaviruses* (includes LVR16A), which cluster on the top, and *Tequintaviruses*, at the bottom. The type species of each genus are marked with a star. The numbers above the branches are GBDP pseudo-bootstrap support values (100 replications). The branch lengths are scaled in terms of the used formula D0.

In addition to T5, we identified several other closely related viruses based on similarity searches (Table S4), including among others the siphophage Stitch [56], *Salmonella* phage Shivani [57], *Escherichia* phage vB_EcoS_FFH1 [58], *Salmonella* phage OSY-STA [59], *Salmonella* phage Seabear [60], *Salmonella* phage Seafire [61], *Salmonella* phage STG2 [62], and *Escherichia* phage phiLLS [63]. The similarity between bacteriophage T5, AFKV33, EPS7, and SPC35 has been demonstrated before [64], as well as between DT57C and T5 [65]. A phylogenetic analysis of several related bacteriophages (Table S4) suggests that ϕR2-01 clusters more closely to epseptimaviruses, such as EPS7 and Stitch, while tequintaviruses form two distinct clusters at whole-genome-level nucleotide analysis (Figure 3 and Figure S2).

### 3.4. Mass Spectrometric Identification of ϕR2-01 Proteins

**Figure 4:**
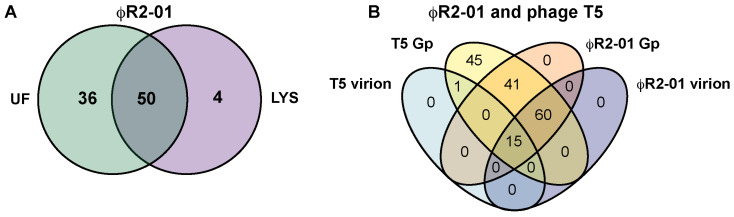
Mass spectrometric characterization of the ϕR2-01 virions and the host cell lysate, and comparison to bacteriophage T5. (**A**) ϕR2-01-specific proteins identified in samples. We identified a total of 90 ϕR2-01-specific proteins, of which 50 were detected both in the ϕR2-01 virion (ultracentrifuged phage, UF) and in the host cell lysate (LYS), whereas an additional 36 proteins were virion-specific and 4 were only detected in the LYS sample. (**B**) Comparison of the bacteriophage-T5-Gp- and bacteriophage-T5-expressed proteins to those homologous on the ϕR2-01 genome, and expressed homologous proteins identified in the ϕR2-01 virion. We describe 15 expressed proteins shared by the two viruses, and an additional 60 ϕR2-01 virion-specific proteins that have homologous counterparts in the T5 genome.

We used in-solution tryptic digestion of ϕR2-01 virions purified by ultracentrifugation as well as a ϕR2-01-infected *Y. enterocolitica* serotype O:8 strain, 8081-c-R2 [28], host cell lysate to identify expressed viral proteins associated with the virion and required during host infection and lysis. By using label-free data-dependent acquisition (DDA) quantification and two different in-house generated datasets for peptide matching we identified, altogether, 90 viral and 878 host-derived proteins (Figure 4A, Tables S5 and S6). One of the in-house generated datasets contained the ϕR2-01 and *Y. enterocolitica* serotype O:8 strain 8081 reference proteomes, and the other dataset contained all six frame translations of the ϕR2-01 genome, yielding 542 ORFs. This approach allowed us to identify two new genes (*g18.1* and *g54.1*) that were missed in the original genome sequence annotation. Notably, this approach could be applied more broadly in mass-spectrometry-based proteome characterization of small viral proteins in order to identify novel, previously unidentified translated ORFs. In summary, a total of 50 of the identified proteins were detected both as a part of the phage particle as well as present in the host cell lysate, whereas 36 proteins were exclusively PPAPs and four were exclusively found in the host cell lysate (assuming the inclusion threshold above) (Figure 4B, Tables S5 and S6). Importantly, our results confirmed the expression of more than 30 uncharacterized proteins (Table S3), warranting further biochemical characterization of these and their role in virus replication and assembly.

Based on label-free spectral counts, the ϕR2-01-encoded PPAPs can be grouped into major virion constituents (average spectral count value > 100), less abundant virion proteins (average spectral count value between 10 and 100), and minor virion components (average spectral count value < 10) based on the measured spectral intensities for this dataset. The major virion-associated proteins are Gp134 (major tail protein, T5.145), Gp130 (tape measure protein, T5.140), Gp140 (head protein, T5.151), Gp138 (major head protein precursor, T5.149), Gp110 (D5 protein, T5.118), Gp141 (portal protein, T5.152), and Gp128 (tail protein, T5.138). Of these, all but the D5 protein have been identified as a part of the T5 virion as well [17]. Proteomic analysis of the T5 phage particles has identified 16 proteins to be virion-associated [17]. Fifteen counterparts of these were found in ϕR2-01 (Figure 4B, Table S7). The one T5 protein missing in ϕR2-01 is protein T5.136 (pb2), which stabilizes the tail sheath structure and acts as a connector between the end of the tail and the portal vertex of the capsid [67]. The corresponding gene is likewise absent in ϕR2-01. Other interesting differences at the genomic level are the ϕR2-01 genes missing in T5, such as ϕR2-01 gene *g123*, encoding an L-shaped tail fiber which is also present in enterobacteria phage DT571/2 [65], and the ϕR2-01 gene *g124*, encoding a predicted tail assembly chaperone (Table S3). Finally, the 1226 amino acid residue length of the tail tape measure protein, Gp130, reflects a tail length of 185 nm perfectly when applying the rule that each amino acid residue contributes 1.5 Å to the tail length [68,69].

### 3.5. ϕR2-01 Host Range and Growth Characteristics

To study the host range of ϕR2-01, 126 *Yersinia* strains, representing 13 *Yersinia* species and several different sero- and biotypes, were tested for sensitivity using the double-layer soft-agar droplet method. Of the 93 *Y. enterocolitica* strains, mostly of human origin (Table S2), 78 were sensitive and only 15 were resistant. Among the 12 other *Yersinia* species only one *Y. kristensenii* serotype O:3 strain was sensitive (Table 2). In addition, fifteen *E. coli* isolates, nine *Salmonella* isolates, and one *Shigella* isolate from the lab collection were tested for ϕR2-01 sensitivity and were all found to be resistant (Table S2). In order to elucidate the role of LPS in adsorption, we tested the sensitivity of several different LPS mutants of *Y. enterocolitica* serotypes O:3 and O:8 (Table 3). All the LPS mutants missing either O-antigens or parts of (or the whole of) the core oligosaccharide were sensitive to the phage, indicating that LPS apparently does not function as a receptor. On the contrary, the EOP determinations indicated that the O-ag substitutions in the serotype O:3 or O:8 LPSs had a blocking effect on adsorption (Table 3).

### 3.6. BtuB Is the ϕR2-01 Host Receptor

**Figure 5:**
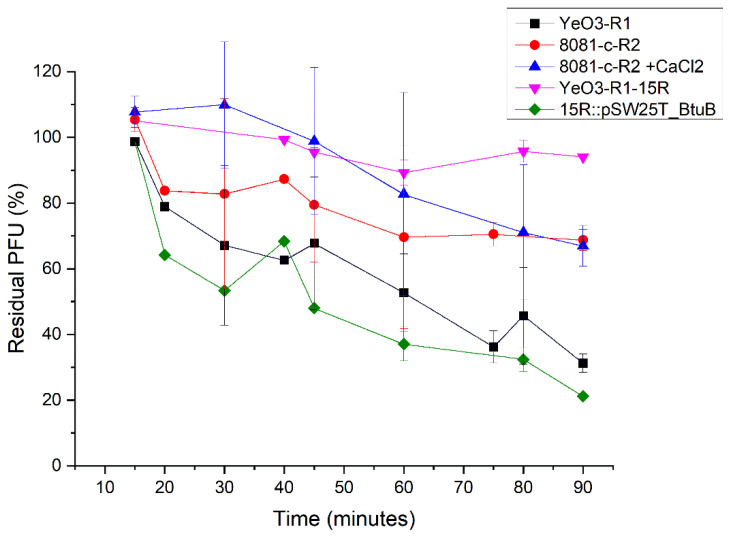
Adsorption kinetics of ϕR2-01 at room temperature. The measurements at time points 30, 45, 60, 75, and 90 min were carried out in quadruplicates, and at other time points only in duplicates or even as single measurements. Adsorption is presented as residual PFU percentage (phage remaining in the supernatant) when the control supernatant without bacteria in each time point was set to 100%. Error bars represent SD between replicates. Strain 8081-c-R2 is the original host strain for phage ϕR2-01. The adsorption kinetics are very slow and not dependent on LPS (or 1 mM of CaCl_2_). Strain YeO3-R1 was used as a control as the transposon mutant YeO3-R1-15R (BtuB knock-out) and its cis-complemented counterpart (YeO3-R1-15R::pSW25T_BtuB) were on YeO3-R1 background.

Due to the adsorption strategy used by T5-like phages, we anticipated that ϕR2-01 would use a similar dual receptor strategy, i.e., both LPS and an outer membrane protein as receptors. However, for ϕR2-01 LPS seemed not to play any role as a receptor, in fact, the presence of the O-polysaccharide (O-antigen, O-ag) seemed to block the receptor, as the EOPs of all the smooth strains were lower than that of rough strains that were all equally susceptible to the phage (Table 3). We therefore screened a transposon insertion mutant library of *Y. enterocolitica* serotype O:3 strain YeO3-R1 [46] for phage-resistant mutants to identify the host receptor. Using this approach, 14 phage-resistant transposon insertion mutants were isolated. Four of these mutants were further analyzed by sequencing to identify the transposon insertion site (Table 1) and the inactivated gene conferring the phage resistance. The insertion site in all the mutants resided at different positions within the *btuB* gene encoding BtuB, indicating that the mutants were independent. To confirm that BtuB indeed is the receptor, we complemented the *Y. enterocolitica*-resistant mutant with a functional *btuB* gene (Table 1). Introduction of a wild-type *btuB* gene into the *btuB* mutant complemented and fully restored the phage sensitivity and adsorption properties of the mutant strain (Table 3 and Figure 5). Furthermore, the adsorption experiments showed that the unusually slow adsorption kinetics are not dependent on calcium, in contrast to what has been demonstrated for, e.g., *Lactobacillus* virulent phage P1, where the addition of calcium ions has been demonstrated to promote and increase the adsorption capacity of P1 [70]. Even though adsorption times of up to 45 min have been reported for some other *Lactobacillus* phages [71], the continued adsorption of ϕR2-01 up until 90 min is uncommon. The cause of this is unknown to date, and requires further investigation beyond the scope of this paper; however, we postulate that this is most likely due to low affinity between the tail fiber proteins and BtuB, allowing for fluctuation in ϕR2-01 attachment to the host. As additional evidence for BtuB, the introduction of the *btuB* gene of *Y. enterocolitica* in plasmids pTM100_BtuB and pSW25T_BtuB (Table 1) to *E. coli* ω7249 converted the strain sensitive to ϕR2-01 (Table 3). These results confirmed conclusively that BtuB is the ϕR2-01 receptor, and most likely targeted by the Gp144 that corresponds to the T5 pb5. These findings are further strengthened by the fact that BtuB co-purifies from the phage lysate with the phage particles during virus preparation, while BtuB could not be identified from the host cell control lysate (Tables S5 and S6). The fact that we cannot identify BtuB in the host cell lysate but only associated with the virions could be due to the fact that BtuB might be a low-abundance protein, which is enriched to the level of detection upon binding to ϕR2-01. Interestingly, in addition to ϕR2-01, the phages EPS7 [72], SPC35 [73], DT57C, and DT571/2, as well as BF23 [74], use BtuB as a host receptor.

The sequence alignment of the BtuB proteins from several sensitive and naturally resistant *Y. enterocolitica* host strains did not show any apparent global differences to the BtuB sequences of the resistant strains. However, when comparing the different BtuB loop regions, it is evident that there is a difference in the EL-7 loop sequences in the sensitive strains as opposed to the ϕR2-01-resistant strains (Figure S3A). The sensitive strains have a consensus sequence of DYSFDNST/IFKG, and the resistant ones a somewhat less conserved sequence of DYXSDPXTXXG (Figure S3A). The BtuB sequences of the phage sensitive strains included in the analysis (i.e., 8081-c-R2 and YeO3) did, furthermore, cluster together in the generated phylogeny tree (Figure S3B). We postulate that the host sensitivity or resistance to ϕR2-01 infection might in part be dependent on the structure of the respective BtuB proteins and in particular the EL-7 loop, but other factors might additionally affect infection. 

**Figure 6:**
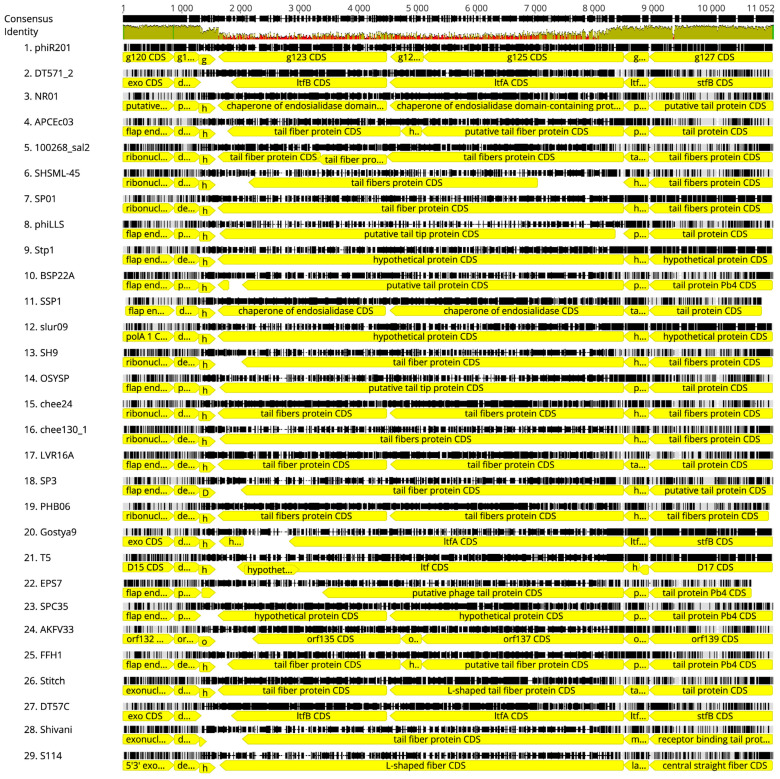
Alignment of the tail fiber gene region from *g120* to *g127*of ϕR2-01 and the related Markadamvirinae (Table S4). The genes are represented by yellow arrows. A green area in the consensus identity indicates high similarity, and a red area indicates low similarity between the three phages. Generated with Geneious v11.1.5 [76] (accessed on 10 October 2020).

For phages DT57C and DT571/2, it has been suggested that the receptor recognition is not responsible for observed host range differences [75]. Interestingly, ϕR2-01 has the same kind of genomic arrangement of tail fibers as DT57C, DT571/2 [75], and other T5-related phages; instead of having one long tail fiber (LTF), as is the case with T5 (T5.133 or pb1) [17], they encode for two shorter tail fibers (Figure 6). In ϕR2-01, these are encoded by the *g123* and *g125* genes, and their expression was verified by proteomics (Table S3). If and how this dual-tail-fiber arrangement of the T5-related phages, including ϕR2-01, is the explanation for the receptor not conferring host range specificity, remains to be determined, as does the exact role of the BtuB EL-7 sequence divergence between ϕR2-01-sensitive and -resistant strains.

Common to BF23 and T5 is that the gene encoding the phage-encoded phage-resistance-conferring lipoprotein involved in host receptor blocking is located directly upstream of the gene encoding the receptor-binding protein [77,78]. This genetic receptor-binding/receptor-blocking module appears to be inherited only as an entity [78], and might be more widespread in T5-type bacteriophages in general. In ϕR2-01, a lipoprotein with a similar function could be encoded by *g145*, which, similar to T5 and BF23, is encoded next to *g144* but in the opposite direction, and has been described as a hallmark of these genetic receptor-binding/receptor-blocking modules [77,78]. Further biochemical characterization combined with infection assays are needed to verify the role of *g145* in host receptor-blocking and subsequent prevention of superinfection. Notably, we did not find Gp145 via mass-spectrometry-based proteomics (Table S3). This might reflect a possible time-course-dependent expression of *g145*.

## 4. Conclusions

Lytic bacteriophages are powerful tools to be used in phage therapy and as biocontrol agents. For *Y. enterocolitica-*mediated food-borne infections the latter would be especially crucial, as it can proliferate at 4 °C, making it dangerous even if food products are stored under refrigeration. A handful of bacteriophages have been described with potential as biocontrol agents to reduce the number of *Y. enterocolitica* colonies in meat [24], food and kitchenware [6], and poultry [25]. Here, we characterize the yersiniophage, ϕR2-01, with a specificity for a broad range of *Y. enterocolitica* strains of different serotypes, therefore apparently irrespective of their LPS structures. As regulatory issues regarding the use of phages as biocontrol agents in the food industry could be the major obstacle, further studies on ϕR2-01 are needed to verify its safety and efficacy as a biocontrol agent.

## Figures and Tables

**Table 1:** List of bacterial strains and plasmids used in the isolation of phage, phage-resistant mutants, complementation, phage adsorption assay and efficiency of plating (EOP) experiments.

Strains	Comment	Reference
Yersinia enterocolitica		
8081-c	Serotype O:8, virulence plasmid cured, smooth	[30]
8081-c-R2	Rough, virulence plasmid cured. Host strain for phage ϕR2-01	[28,31]
6471/76-c (YeO3-c)	Serotype O:3, virulence plasmid cured	[32]
YeO3-R1 (=YeO3-c-R1)	Spontaneous rough derivative of 6471/76-c	[33]
YeO3-c-OC	∆(*wzx-wbcQ*), derivative of 6471/76-c	[34]
YeO3-c-OCR	Spontaneous rough derivative of YeO3-c-OC	[34]
YeO3-c-R1-M164	*waaF*::Cat-Mu derivative of YeO3-c-R1	[35]
YeO3-c-R1-M196	*galU*::Cat-Mu derivative of YeO3-c-R1	[35]
YeO3-c-R1-M205	*hldE*::Cat-Mu derivative of YeO3-c-R1	[35]
YeO3-R1-10R	*btuB*::Cat-Mu with insertion at position 2,853,209 of Y11 genome *	This work
YeO3-R1-11R	*btuB*::Cat-Mu with insertion at position 2,853,177 of Y11 genome *	This work
YeO3-R1-15R	*btuB*::Cat-Mu with insertion at position 2,853,689 of Y11 genome *	This work
YeO3-R1-20R	*btuB*::Cat-Mu with insertion at position 2,852,224 of Y11 genome *	This work
YeO3-R1-15R::pSW25T_BtuB	Cis-complemented *btuB*::Cat-Mu strain	This work
*Escherichia coli*		
DH10B	Used for cloning and plasmid isolation	Invitrogen
ω7249	Host for suicide plasmids (chrRP4Δnic35)	[36]
Plasmids		
pTM100	TetR	[37]
pTM100_BtuB	Complementation plasmid with wild-type *btuB* gene cloned into pTM100	This work
pSW25T	Mobilizable suicide plasmid, SpecR	[38]
pSW25T_BtuB	Complementation suicide plasmid with wild-type *btuB* gene cloned into pSW25T	This work

* Y11 genomic location encodes for outer membrane vitamin B12 receptor BtuB (CBY25391.1). YeO3-R1::Cat-Mu phage-resistant transposon mutants were sequenced using arbitrary PCR and MucInt primer as described in the Materials and Methods section. The host strain for phage ϕR2-01 is 8081-c-R2.

**Table 2:** Bacteriophage ϕR2-01 sensitivity of *Yersinia* species ^a^.

*Yersinia* Species	Phage-Sensitive Serotypes ^b^	Serotypes with Phage-Sensitive (S) and -Resistant (R) Strains	Phage-Resistant Serotypes ^c^
*Y. enterocolitica*	O:1 [2], O:2 [2], O:3 [11], O:4 [1], O:4,32 [1], O:5 [7], O:5,27 [5], O:6 [2], O:6,31 [2], O:7,8 [2], O:8 [12], O:9 [8], O:13 [1], *O:13a,13b* [1] ^d^, O:13,7 [2], O:13,18 [1], O:14 [1], O:20 [2], O:21 [3], O:25 [1], O:34 [1], O:35,36 [1], O:35,52 [1], *O:41(27),K1* [1], *O:41(27),42* [1], O:41,43 [1], and O:50 [1]	*O:6,30* [1S/2R], *O:10* [1S/3R], and *O:41(27),43* [1S/1R]	*O:1,2,3* [1] *O:25,26,44* [1], *O:26,44* [1], *O:28,50* [1], *O:41(27),42,K1* [1] ^e^, *K1 NT* [2], and *NT* [2]
*Y. aleksiciae*			O:16 [2]
*Y. bercovieri*			O:58,16 [2], NT [1]
*Y. frederiksenii*			O:3 [1], O:16 [1], O:35 [1], O:48 [1], K1 NT [1], NK [2], and NT [1]
*Y. intermedia*			O:16,21 [1], O:52,54 [1], and NK [1]
*Y. kristensenii*	*O:3* [1]		O:12,15 [1], NT [1], and UT [1]
*Y. mollaretii*			O:3 [1] and O:59(20,36,7) [1]
*Y. nurmii*			UT [1]
*Y. pekkanenii*			NK [1]
*Y. pestis*			NA [2]
*Y. pseudotuberculosis*			O:1b [2] and O:3 [2]
*Y. rohdei*			NK [1]
Y. ruckeri			NK [1] and UT [2]

^a^ Sensitivity was tested for 126 *Yersinia* species strains (see Table S2 in the supplemental material) at RT. ^b^ The number of strains is given in brackets. ^c^ NA, not applicable; NT, non-typeable and either cross-reacting or not agglutinating with *Y. enterocolitica* O:3, O:5, O:8, or O:9 antisera; NK, not known; and UT, untyped. ^d^ Strains with sequenced *btuB* genes are indicted in italics and in Table S2. ^e^ This strain (#729 in Table S2) failed PCR amplification and is thus not included in the final analysis.

**Table 3:** Bacteriophage ϕR2-01 sensitivity presented as efficiencies of plating (EOP) in bacterial host strains with different LPS phenotypes. The original isolation host for ϕR2-01 is 8081-c-R2.

Strain	LPS Composition *	EOP with ϕR2-01
8081-c	LA-IC-O-ag (smooth)	1 × 10^−6^
8081-c-R2	LA-IC (rough)	1
6471/76-c (YeO3-c)	LA-IC-OC-O-ag (smooth)	0.1
YeO3-R1 (=YeO3-c-R1)	LA-IC-OC (rough)	1
YeO3-c-OC	LA-IC-O-ag (smooth)	0.02
YeO3-c-OCR	LA-IC (rough)	1
YeO3-c-R1-M196	LA-Rd1 (deep rough)	1
YeO3-c-R1-M164	LA-Rd2 (deeper rough)	1
YeO3-c-R1-M205	LA-Re (deepest rough)	1
YeO3-R1-15R	LA-IC-OC (rough), *btuB::CatMu* mutant	0
YeO3-R1-15R::pSW25T_BtuB	LA-IC-OC (rough), *btuB-*complemented	1
YeO3-R1-15R/pTM100_BtuB	LA-IC-OC (rough), *btuB-*complemented	1
ω7249	*E. coli* K12 (rough)	0
ω7249/pTM100_BtuB	*E. coli* K12 (rough), expresses Ye BtuB	1

* LA, lipid-A; IC, inner core; OC, outer core; and O-ag, O-antigen. Of the mutants, YeO3-R1-M205 had only the Kdo residues of the IC present (Re type), and the two other mutants YeO3-R1-M196 and YeO3-R1-M164 had less truncated ICs (Rd1 and Rd2 types, respectively) [35]. The host strain for phage ϕR2-01 is 8081-c-R2.

## Data Availability

The genome sequence of the *Yersinia* phage ϕR2-01 is available in GenBank under the accession number HE956708. The sequences of the *btuB* genes of the *Yersinia* strains sequenced in this work have been deposited to GenBank under the accession numbers OK169486-OK169508. The mass spectrometry data have been deposited to the ProteomeXchange [79] consortium via the MassIVE partner repository (https://massive.ucsd.edu/ (accessed on 19 June 2021)) with the dataset identifiers PXD009346 (reference proteomes) and PXD009347 (six-frame translation of the ϕR2-01 genome).

## Supplementary Materials

The following are available online at https://www.mdpi.com/article/10.3390/v13112171/s1. Figure S1. The pSW25T_BtuB suicide plasmid. Figure S2. Whole-genome nucleotide sequence alignment of ϕR2-01 and 28 related phages. Figure S3. Sequence alignment of BtuB proteins and the Yersinia BtuB phylogeny tree. Table S1. Primers used in this study. Table S2. List of bacterial strains used in phage host range experiments. Table S3. Genome annotation of phage ϕR2-01. Table S4. Phage genome sequences used for Victor and Clustal multiple sequence alignment. Table S5. ϕR2-01 and *Y. enterocolitica* strain 8081 proteins identified that are associated with the virion. Table S6. ϕR2-01 and host proteins identified in the host cell lysate. Table S7. Comparison between ϕR2-01 and bacteriophage T5 genes and virion-associated proteins.
